# Comparative sera proteomics analysis of differentially expressed proteins in oral squamous cell carcinoma

**DOI:** 10.7717/peerj.11548

**Published:** 2021-06-10

**Authors:** Yin-Ling Wong, Anand Ramanathan, Kar Mun Yuen, Wan Mahadzir Wan Mustafa, Mannil Thomas Abraham, Keng Kiong Tay, Zainal Ariff Abdul Rahman, Yeng Chen

**Affiliations:** 1 Department of Oral & Craniofacial Sciences, Faculty of Dentistry, University of Malaya, Kuala Lumpur, Malaysia; 2 Department of Oral & Maxillofacial Clinical Sciences, Faculty of Dentistry, University of Malaya, Kuala Lumpur, Malaysia; 3 Oral Cancer Research & Coordinating Centre, Faculty of Dentistry, University of Malaya, Kuala Lumpur, Malaysia; 4 Oral Health Program, Ministry of Health, Federal Government Administrative Centre, Putrajaya, Malaysia; 5 Faculty of Dentistry, MAHSA University, Jenjarum, Selangor, Malaysia

**Keywords:** Biomarker, Proteomics, Oral potentially malignant disorder, Oral squamous cell carcinoma

## Abstract

**Background:**

Oral squamous cell carcinoma (OSCC) has increased in incidence from 1990 to 2017, especially in South and Southeast Asia. It is often diagnosed at an advanced stage with a poor prognosis. Therefore, early detection of OSCC is essential to improve the prognosis of OSCC. This study aims to identify the differentially expressed serum proteins as potential biomarkers for oral squamous cell carcinoma (OSCC).

**Methods:**

Comparative proteomics profiling of serum samples from OSCC patients, oral potentially malignant disorder (OPMD) patients, and healthy individuals were performed using two-dimensional gel electrophoresis (2-DE) coupled with mass spectrometry (MS) (*n* = 60) and bioinformatics analysis. The enzyme-linked immunosorbent assay (ELISA) (*n* = 120) and immunohistochemistry (IHC) (*n* = 70) were used to confirm our findings.

**Results:**

The 2-DE analysis revealed that 20 differentially expressed proteins were detected in OPMD and OSCC (*p* < 0.05). Bioinformatics analysis indicated that the activation of classical complement, liver X receptor/retinoid X receptor (LXR/RXR) activation, and acute phase response signaling pathway are associated with the development and progression of OSCC. Most of the detected proteins are acute-phase proteins and were related to inflammation and immune responses, including apolipoprotein A-I (APOA1), complement C3 (C3), clusterin (CLU), and haptoglobin (HP). The expression levels of CLU and HP in ELISA are consistent with the findings from the 2-DE analysis, except for the mean serum level of HP in OPMD, whereby it was slightly higher than that in control. IHC results demonstrated that CLU and HP are significantly decreased in OSCC tissues.

**Conclusion:**

Decreased expression of CLU and HP could serve as complementary biomarkers of OSCC. These proteins may assist in predicting the outcomes of OSCC patients. However, a larger cohort is needed for further investigation.

## Introduction

Oral squamous cell carcinoma (OSCC) is one of the commonly diagnosed head and neck cancers worldwide (Siegel, Miller & Jemal, 2020; Vigneswaran & Williams, 2014). The incidence of OSCC is increased from 1990 to 2017, especially in South and Southeast Asia regions (Du et al., 2020). OSCC can affect any anatomic sites within the oral cavity, in which it is commonly found in the tongue, gingiva, and floor of the mouth (Pires et al., 2013). OSCC usually arises from apparently normal oral epithelium, but it also can progress from potentially malignant states without any specific symptoms (Markopoulos, 2012). It is widely known that tobacco smoking, alcohol drinking, and betel quid chewing are the risk factors of OSCC (Kumar et al., 2016). Intriguingly, oral potentially malignant disorder (OPMD) and OSCC have similar risk factors (Johnson, Jayasekara & Amarasinghe, 2011).

The current approaches for OSCC and OPMD screening are mainly the conventional oral examination followed by biopsy of suspicious tissues with the histopathological assessment (Carreras-Torras & Gay-Escoda, 2015). However, the efficacy of these approaches remains controversial, and it may cause a delay in the diagnosis since the early stage of OSCC has frequently been reported to be asymptomatic. Thus, OSCC is mostly diagnosed at an advanced stage, which has an aggressive clinical behavior and poor prognosis. Despite the advancement in diagnosis and treatment modalities, the five-year survival rate of OSCC is less than 50% (Le Campion et al., 2017). A comprehensive insight on the biological characteristics and mechanisms in the development and progression of OSCC would lead to the discovery of biomarkers that may facilitate earlier diagnosis, therefore improving the prognosis of OSCC patients.

Oral carcinogenesis is a multistep process involving molecular and histological changes, and this had made OSCC is a complex and heterogeneous disease. Comparative proteomics studies could be used to reveal the pathogenesis of diseases and discover the potential protein biomarkers. Numerous proteomics studies have been conducted to identify potential biomarkers of OSCC for diagnosis, prognosis, and treatment purposes (Chen et al., 2014; Csosz et al., 2018; Ni et al., 2015; Tung et al., 2013; Yu et al., 2011). This has shown that proteomics technology is essential in the analysis of non-invasive or minimally invasive biomarkers’ resources, including serum, plasma, and saliva, for their clinical utility in OSCC.

In the present study, a proteomics approach was employed to analyze the serum protein profile of OSCC patients. It is known that serum contains a mixture of proteins with a wide dynamic range. When depletion strategy is applied to remove high abundant proteins in serum, it may induce bias that can impair technical reproducibility and cause the loss of some non-targeted proteins that might interact strongly with the high abundant proteins (Bellei et al., 2011; Tu et al., 2010). Therefore, two-dimensional gel electrophoresis (2-DE) and mass spectrometry (MS) for the identification of differentially expressed proteins as potential serum biomarkers without removal of high abundant proteins was carried out in this study. Additionally, the differentially expressed proteins were assessed using functional annotation and pathway analysis. The candidate biomarkers were validated by enzyme-linked immunosorbent assay (ELISA) and immunohistochemistry (IHC) methods.

## Materials & Methods

### Samples collection

The serum and formalin-fixed paraffin-embedded (FFPE) tissue samples of OSCC were obtained from the Malaysian Oral Cancer Database and Tissue Bank System (MOCDTBS) coordinated by the Oral Cancer Research & Coordinating Centre, University of Malaya (OCRCC-UM) {Medical Ethics Approval Number: DFOP1504/0084(L)} following approval by the Ethics Committee of the Faculty of Dentistry, University of Malaya {Medical Ethics Approval Number: DFOC1803/0026(P)}. These samples were acquired from patients with a histologically confirmed diagnosis of OSCC. No patients received radio- or chemotherapy prior to surgery. OPMD samples were also included in this study as OPMDs have been associated with a risk of malignant transformation to OSCC (Warnakulasuriya, Johnson & Waal, 2007). For the control group, serum samples were obtained from healthy individuals, while FFPE tissue samples were obtained from subjects who are visiting the clinic for removal of impacted wisdom teeth with informed consent. These control subjects did not have a previous history of OSCC or other cancers.

Serum samples consisted of early OSCC (*n* = 20), advanced OSCC (*n* = 20), OPMD (*n* = 10), and normal control (*n* = 10) were used for the proteomics analysis. For validation purposes, antibody-based methods were conducted in a larger cohort. A total of 120 serum samples consisted of OSCC patients from early stage (*n* = 34) and advanced stage (*n* = 39), OPMD patients (*n* = 12), and healthy individuals (*n* = 35) were used for ELISA analysis. Out of the 120 serum samples, 60 samples are from the proteomics study set. Briefly, the blood samples (3–4 ml) were collected into BD Vacutainer Plus plastic serum tubes that containing increased silica act clot activator and silicone-coated interior (Becton Dickinson, Heidelberg, Germany) by trained phlebotomists. The blood samples were collected and processed in accordance with the established protocol for sample collection as outlined by the International Society for Biological and Environmental Repositories (ISBER) Best Practices for Repositories, Collection, Storage and Retrieval of Human Biological Materials for Research (Klont et al., 2019; Vaught & Henderson, 2011). Upon processing of the blood samples, the serum samples were aliquoted into 300 µl portions and stored at −80 °C until further use. Whereas 70 FFPE tissue samples consisted of early stage OSCC (*n* = 18), advanced stage OSCC (*n* = 21), OPMD (*n* = 11), and normal control (*n* = 10) were included for IHC analysis. The serum and FFPE tissue samples are independent of each other. All analysis of these samples was performed individually, and detailed clinical information of the samples is shown in Tables S1 and S2.

### Two-dimensional gel electrophoresis (2-DE) and Mass Spectrometry (MS) analyses

The 2-DE and MS analyses were performed as previously described using three µl of unfractionated serum samples with an estimated protein concentration of 150 µg (Chen et al., 2014). The first-dimensional of the 2-DE analysis was carried out on 11 cm, pH 4–7 L Immobiline DryStrip gels (GE Healthcare Biosciences, Uppsala, Sweden). This was followed by the second-dimensional separation with 12.5% sodium dodecyl sulfate-polyacrylamide gel electrophoresis (SDS-PAGE) gel using the SE 600 Ruby system (GE Healthcare Biosciences, Uppsala, Sweden). The 2-DE gels were then visualized by silver staining and the images of 2-DE gels were scanned with ImageQuant LAS 500 (GE Healthcare Biosciences, Uppsala, Sweden). After that, the gel images were analyzed using Progenesis SameSpots v4.0 software (Totallab, Newcastle, UK). The protein spots of interest were manually excised from the 2-DE gels and proceeded to in-gel trypsin digestion. The tryptic peptides were then reconstituted in 0.1% formic acid and desalted using ZipTip C18 pipette tips (Millipore, Massachusetts, USA). Subsequently, the eluted peptides were mixed with an equal volume of *α*-cyano-4-hydroxy-cinnamic acid (CHCA) matrix solution (six mg/ml) and spotted onto a MALDI target plate. After air dried, the peptide mixtures were analyzed with a 4,800 Plus MALDI-TOF/TOF Analyzer (AB Sciex, Foster City, CA, USA). Calibration of the instrument was performed using trypsin-digested beta-galactosidase (Mass Standards kit, AB Sciex, Foster City, CA. USA). In-house prepared silver-stained bovine serum albumin (five µg/ml) gel plugs was used as internal control. The MS results were acquired automatically with a trypsin auto-digest exclusion list and 20 most intense precursor ions were selected for tandem mass spectrometry (MS/MS) analysis with a minimum signal-to-noise (S/N) of at least 10. The protein identification was conducted using the MASCOT search engine (Matrix Science, London, UK) against all human proteins and sequence information in the UniProtKB database (last updated: December 2016). Search parameters for the MASCOT peptide mass fingerprint were set as a fixed modification on carbamidomethylation of cysteines, variable modification of methionine oxidation, up to one missed tryptic cleavage is allowed, MS precursor ion mass tolerance at 100 ppm, and MS/MS fragment ion mass tolerance of ±0.2 Da. A MASCOT score greater than 50 were considered significant (*p* < 0.05).

### Functional classification, annotation and pathway analyses

The identified proteins were further determined using Protein ANnotation Through Evolutionary Relationships (PANTHER) v16.0 (http://www.pantherdb.org/) and Database for Annotation, Visualization, and Integrated Discovery (DAVID) v6.8 (https://david.ncifcrf.gov/home.jsp) for functional classification and annotation analysis. Both PANTHER and DAVID are bioinformatics tools that enable functional protein grouping and annotation using gene ontology terms to understand the biological importance associated with a list of genes or proteins of interest. The proteins were also subjected to pathway analysis using Ingenuity Pathway Analysis (IPA) v7.1 (Qiagen Ingenuity System, California, USA) software to explore the pathway annotations and interaction networks. The interaction networks and predominant canonical pathways of the proteins were generated algorithmically using the Ingenuity pathways knowledge base.

### Enzyme-linked Immunosorbent Assay (ELISA)

A quantitative sandwich ELISA technique was used to determine the serum concentrations of clusterin (CLU) and haptoglobin (HP) in this study. The serum samples were analyzed in duplicate using commercially available CLU and HP ELISA kits (Cusabio Biotech, Wuhan, China) according to the manufacturer’s protocol. Once the chromogenic reaction was stopped with stop solution, the optical density (OD) value was measured at absorbance 450 nm. After that, the concentration of CLU and HP in the serum samples was determined by assessing the OD value of the samples against the respective standard curve.

### Immunohistochemistry (IHC)

For immunohistochemical analysis, the FFPE tissue sections were dewaxed with xylene and rehydrated with gradient ethanol prior to heat-induced antigen retrieval. The peroxidase-blocking solution (Dako, Agilent, Santa Clara, CA, USA) and background sniper (Biocare Medical, Concord, CA, USA) were employed to quench the endogenous peroxidase activity and non-specific background, respectively. Subsequently, the sections were incubated with the diluted primary antibodies: CLU (1:400 dilution, ab92458, Abcam, Cambridge, MA, USA), and HP (1:160 dilution, ab23100, Abcam, Cambridge, MA, USA) for an hour at room temperature. Secondary antibody and 3,3′-diaminobenzidine solution (DAB) chromogen substrate were then applied according to the manufacturer’s protocol (Dako Real EnVision Detection System and Peroxidase/DAB+, Agilent, Santa Clara, CA, USA). After counterstained with hematoxylin, the sections were dehydrated and mounted.

### Statistical analysis

Statistical analysis was performed using SPSS 20.0 statistical software package (SPSS Inc, Chicago, IL, USA). The data of ELISA are exhibited as mean ± standard error mean (SEM). Whereas IHC data are presented as immunoreactive scores (IRS). The immunoreactive scores (IRS) were calculated based on the intensity scores (negative = 0, mild = 1, moderate = 2, and strong = 3) and the percentage of immunopositive staining (0 = ≤10%, 1 = 11–25%, 2 = 26–50%, 3 = 51–75%, and 4 = >75%), whereby the scores were determined by multiplying the intensity and percentage of immunopositive staining scores. Receiver operator characteristic (ROC) curve and logistic regression analyses were used to evaluate the ELISA and IHC data. Statistical comparisons with *p*-values less than 0.05 were considered statistically significant.

## Results

### Identification of differentially expressed proteins by 2-DE and MS analysis

**Figure 1:**
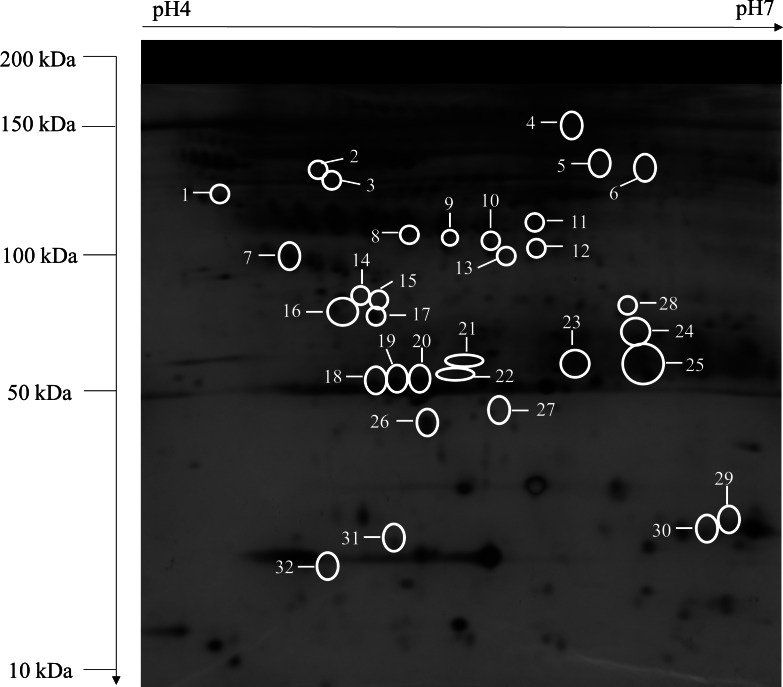
A typical representative of 2-DE serum protein profile (control). Unfractionated serum samples of control, OPMD, early OSCC, and, advanced OSCC were subjected to 2-DE and silver staining. The identified protein spots with differentially expression from control were numbered and circled.

High-resolution serum protein profiles of control, OPMD, early OSCC, and advanced OSCC were obtained from the 2-DE separation and silver staining of the unfractionated serum samples. Thirty-two protein spots that corresponded to 20 differentially expressed proteins showed a statistically significant difference with fold change cut-off of 1.2 between the study groups in the comparative analysis of the gel images (*p* < 0.05, FC ≥ 1.2) (Fig. 1, Table 1). For OPMD, five proteins (AAT, APOA1, IGKC, SAMP, and VDBP) were found to have an increased expression, and the expression levels of 7 proteins (AINX, AMBP, CLU, HP, PRDX2, RAD50, and RBP4) were decreased when compared with controls. In early OSCC, the expression levels of 4 proteins (IGKC, IGHA2, IGHG2, and TF) were increased, and 8 proteins (ALB, AMBP, ARF, CLU, HP, LRG1, RAD50, and VCL) were decreased in expression. Whereas in advanced OSCC, five proteins (AAT, APOA1, C3, IGHG2, and VDBP) were increased in expression, and 2 proteins (ARF and PRDX2) were found to have a decrease in expression when compared with controls.

**Table 1:** MS identification of differentially expressed proteins.

**Spot**	**Protein entry name**	**Protein name**	**UniprotKB accession No.**	**Theoretical mass (kDa)/pI**	**Peptides matched/ sequence coverage (%)**	**Mascot score**	**OPMD vs control**	**Early OSCC vs control**	**Advanced OSCC vs control**
1	LRG1	Leucine-rich alpha-2-glycoprotein	P02750	38.38/6.45	13/23	141	−1.1	**−1.6**	−1.4
2	AAT	*α*1-antitrypsin	P01009	46.88/5.37	21/44	70	**2.2**	1.1	1.4
3	AAT	*α*1-antitrypsin	P01009	46.88/5.37	19/29	82	**1.9**	1.5	**1.5**
4	IGHA2	Ig alpha-2 chain C region	P01877	37.30/5.71	7/12	72	−1.1	**1.7**	1.3
5	TF	Serotransferrin	P02787	79.29/7.66	17/14	71	1.6	**1.9**	1.4
6	IGHG2	Ig gamma-2 chain C region	P01859	36.51/7.65	13/17	60	1.6	**1.8**	**1.6**
7	CLU	Clusterin	P10909	53.03/5.89	11/17	128	**−1.8**	**−1.6**	−1.2
8	HP	Haptoglobin	P00738	45.86/6.13	5/11	41	**−2.5**	−1.0	−1.1
9	HP	Haptoglobin	P00738	45.86/6.13	15/21	293	**−2.8**	−1.2	−1.1
10	HP	Haptoglobin	P00738	45.86/6.13	10/20	177	**−3.4**	−1.2	−1.1
11	HP	Haptoglobin	P00738	45.86/6.13	24/33	225	**−2.6**	**−1.6**	−1.3
12	HP	Haptoglobin	P00738	45.86/6.13	9/20	69	−2.5	−1.1	−1.1
13	AINX	alpha-internexin	Q16352	55.53/5.34	26/41	48	**−2.4**	1.2	−1.0
14	VDBP	Vitamin D-binding protein	P02774	54.53/5.40	15/14	72	**2.3**	1.5	**1.9**
15	C3	Complement C3	P01024	188.57/6.02	31/12	251	−1.1	1.3	**1.5**
16	AMBP	Alpha-1-microglobulin/bikunin precursor	P02760	39.89/5.95	10/29	85	**−2.1**	−1.3	−1.1
17	AMBP	Alpha-1-microglobulin/bikunin precursor	P02760	39.89/5.95	17/28	182	**−2.9**	**−1.9**	1.3
18	APOA1	Apolipoprotein A-I	P02647	30.76/5.56	21/48	249	**2.8**	1.4	**1.6**
19	APOA1	Apolipoprotein A-I	P02647	30.76/5.56	33/62	572	**1.9**	1.2	1.2
20	APOA1	Apolipoprotein A-I	P02647	30.76/5.56	22/46	208	**2.0**	1.2	1.3
21	SAMP	Serum amyloid P-component	P02743	25.49/6.10	5/14	79	**2.0**	1.5	1.3
22	IGKC	Ig kappa chain C region	P01834	11.77/5.58	3/32	93	**2.3**	1.3	1.1
23	IGKC	Ig kappa chain C region	P01834	11.77/5.58	7/54	169	**2.0**	**1.7**	1.4
24	IGKC	Ig kappa chain C region	P01834	11.77/5.58	2/15	41	**1.7**	1.2	1.3
25	IGKC	Ig kappa chain C region	P01834	11.77/5.58	4/32	77	**1.6**	1.2	1.1
26	RBP4	Retinol-binding protein 4	P02753	23.34∕5∕76	11/47	346	**−2.5**	−1.2	−1.3
27	PRDX2	Peroxiredoxin-2	P32119	22.05/5.66	19/60	321	**−2.3**	−1.1	**−1.8**
28	ALB	Albumin	P02768	71.32/5.92	25/24	124	−1.3	**−1.6**	−1.3
29	ALB	Albumin	P02768	71.32/5.92	8/9	56	1.2	**−1.9**	−1.2
30	ARF	Tumour suppressor ARF	Q8N726	14.95/12.41	5/21	17	−1.0	**−2.0**	**−1.6**
31	RAD50	DNA repair protein RAD50	Q92878	154.82/6.48	33/24	42	**−2.0**	**−2.1**	−1.3
32	VCL	Vinculin	P18206	124.29/5.50	54/32	43	−1.9	**−1.9**	−1.3

**Notes.**

Bold: statistically significant difference (adjusted *p* < 0.05) when compared with control.

### Functional classification, annotation and pathway analyses

**Figure 2:**
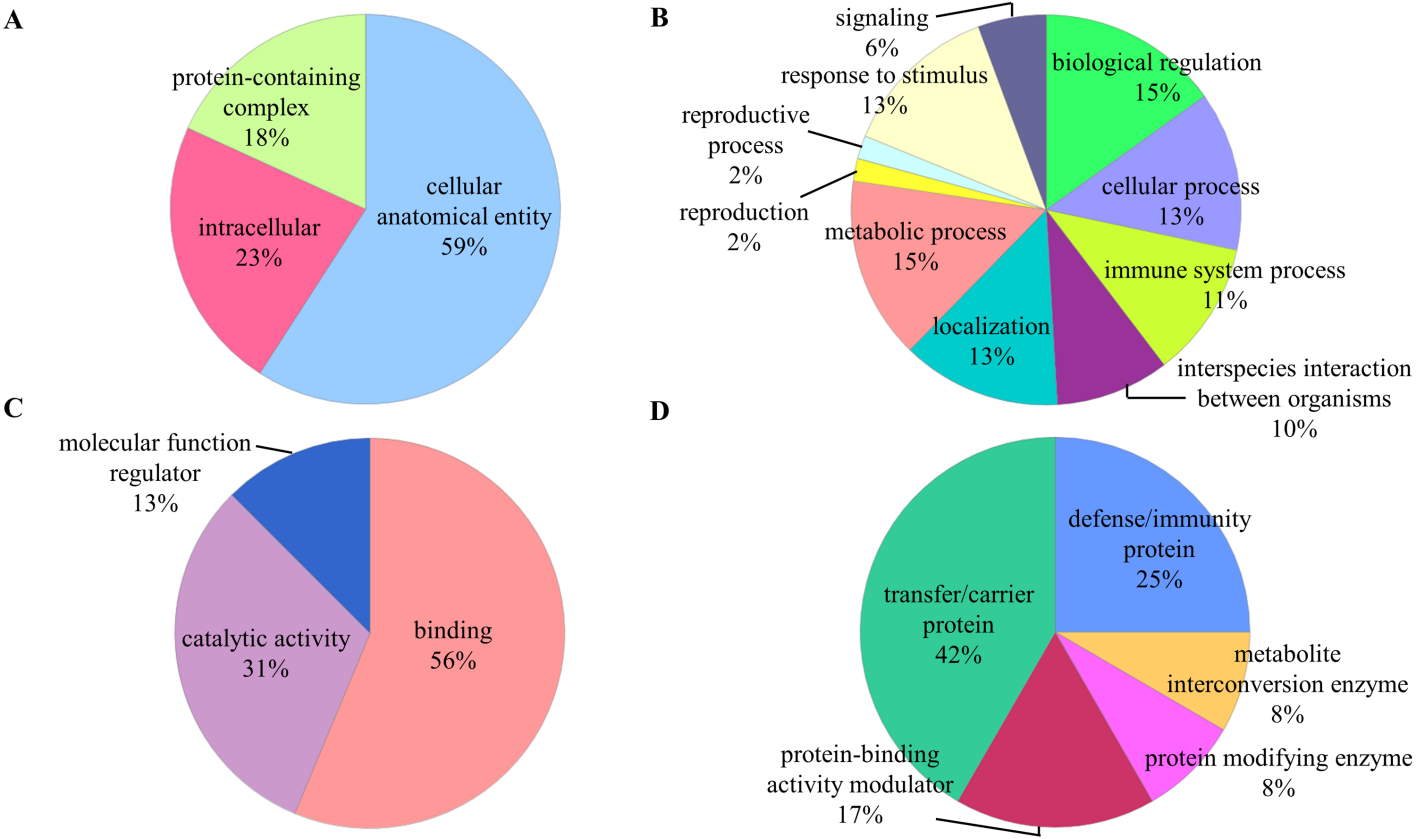
Functional classifications of the identified proteins. Pie charts display classification of the identified proteins regarding to (A) cellular component, (B) biological process, (C) molecular function, and (D) protein class in the PANTHER analysis.

The identified proteins were classified according to their cellular component, biological process, molecular function, and protein class in PANTHER analysis (Fig. 2). In the aspect of cellular component, most of the identified proteins contained cellular anatomical entity (59%). The predominant biological processes of the identified proteins were biological regulation (15%) and metabolic process (15%). These proteins were also involved in binding (59%) and catalytic activity (31%) as most of them are classified as transfer/carrier protein (42%).

The functional annotation clustering analysis revealed that the identified proteins are related to the regulation of cellular processes (Table 2). Most of the identified proteins were enriched within the extracellular space or region. Among these proteins, five proteins (C3, CLU, IGHA2, IGHG2, and IGKC) were implicated in the activation of classical complement pathway. The major molecular function of these proteins was related to catalytic and binding activity, of which 4 proteins (C3, IGHG2, IGKC, and HP) were associated with the serine-type endopeptidase activity.

**Table 2:** Functional annotation analysis of identified proteins using DAVID v6.8.

**GO group**	**Term name**	**Protein count** **(%)**	**Protein entry name**	**Fold enrichment**	*p*-value
**Enrichment score: 14.70**					
Cellular component	Blood microparticle	12 (60.00)	ALB, AMBP, APOA1, C3, CLU, HP, IGHA2, IGHG2, IGKC, SAMP, TF, VDBP	71.94	6.68E−19
Cellular component	Extracellular space	16 (80.00)	AAT, AINX, ALB, AMBP, APOA1, C3, CLU, HP, IGHA2, IGHG2, IGKC, LRG1, RBP4, SAMP, TF, VDBP	10.82	2.91E−14
Cellular component	Extracellular region	16 (80.00)	AAT, ALB, AMBP, APOA1, C3, CLU, HP, IGHA2, IGHG2, IGKC, LRG1, RBP4, SAMP, TF, VCL, VDBP	9.06	4.04E−13
**Enrichment score: 3.21**					
Biological process	Complement activation, classical pathway	5 (25.00)	C3, CLU, IGHA2, IGHG2, IGKC	42.40	4.12E−06
Biological process	Complement activation	4 (20.00)	C3, CLU, IGHG2, IGKC	38.60	1.23E−04
Molecular function	Serine-type endopeptidase activity	4 (20.00)	C3, IGHG2, IGKC, HP	13.24	0.003
**Enrichment score: 3.15**					
Molecular function	Immunoglobulin receptor binding	3 (15.00)	IGHA2, IGHG2, IGKC	97.39	3.84E−04
Biological process	Positive regulation of B cell activation	3 (15.00)	IGHA2, IGHG2, IGKC	96.87	3.88E−04
Biological process	Phagocytosis, recognition	3 (15.00)	IGHA2, IGHG2, IGKC	89.95	4.51E−04
Biological process	Phagocytosis, engulfment	3 (15.00)	IGHA2, IGHG2, IGKC	71.97	7.06E−04
Biological process	Innate immune response	5 (25.00)	CLU, IGHA2, IGHG2, IGKC, VDBP	9.76	0.001
Biological process	B cell receptor signalling pathway	3( (15.00))	IGHA2, IGHG2, IGKC	46.64	0.002
Molecular function	Antigen binding	3(15.00)	IGHA2, IGHG2, IGKC	24.58	0.006
Cellular component	External side of plasma membrane	3 (15.00)	IGHA2, IGHG2, IGKC	12.83	0.020
**Enrichment score: 2.80**					
Biological process	Platelet degranulation	6 (30.00)	AAT, ALB, APOA1, CLU, TF, VCL	48.91	8.55E−08
Cellular component	Platelet alpha granule lumen	3 (15.00)	AAT, ALB, CLU	49.70	0.001

**Figure 3:**
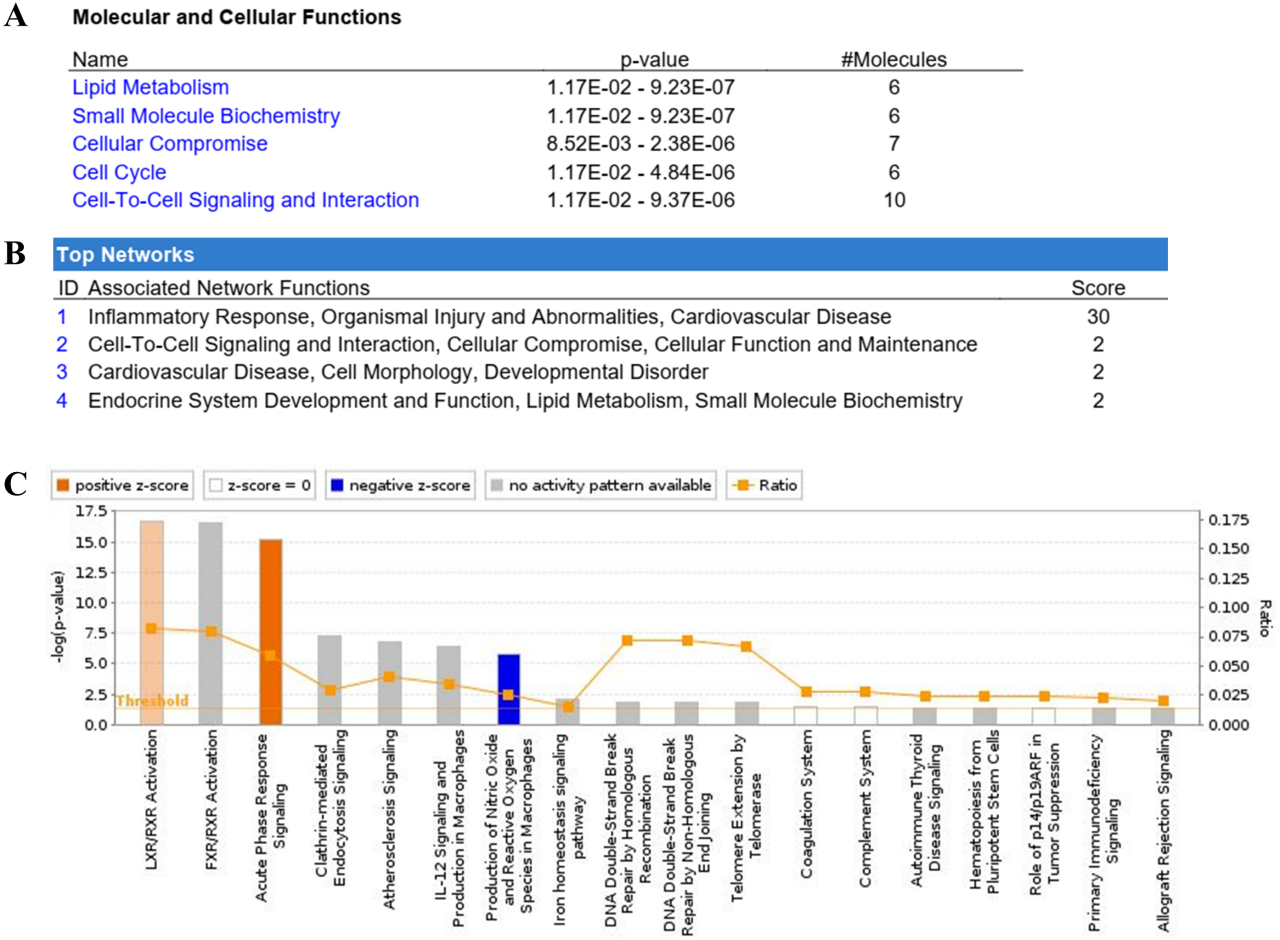
Ingenuity Pathway Analysis (IPA) of identified proteins. The top significantly associated (A) molecular and cellular functions, (B) associated network function, and (C) canonical pathways with the identified proteins.

The IPA showed the pathway annotations and interaction networks between the identified proteins (Fig. 3). For the category of molecular and cellular function, these proteins are involved in lipid metabolism, small molecule biochemistry, cellular compromise, cell cycle, and cell-to-cell signaling and interaction. Whereas for the category of associated network function, the most prominent network of the identified proteins is related to inflammatory response, and organismal injury and abnormalities. The top five canonical pathways in IPA were liver X receptor/retinoid X receptor (LXR/RXR) activation, farnesoid X receptor/retinoid X receptor (FXR/RXR) activation, acute phase response signaling, clathrin-mediated endocytosis signaling, and atherosclerosis signaling. Of these pathways, the LXR/RXR activation and acute phase response signaling pathways are actively associated with OSCC. Nine proteins (AAT, ALB, AMBP, APOA1, C3, CLU, RBP4, TF, and VDBP) from our dataset were found to be involved in the LXR/RXR activation pathway. There were also nine proteins (AAT, ALB, AMBP, APOA1, C3, HP, RBP4, SAMP, and TF) that are associated with the acute phase response signaling pathway.

### ELISA analysis

**Figure 4:**
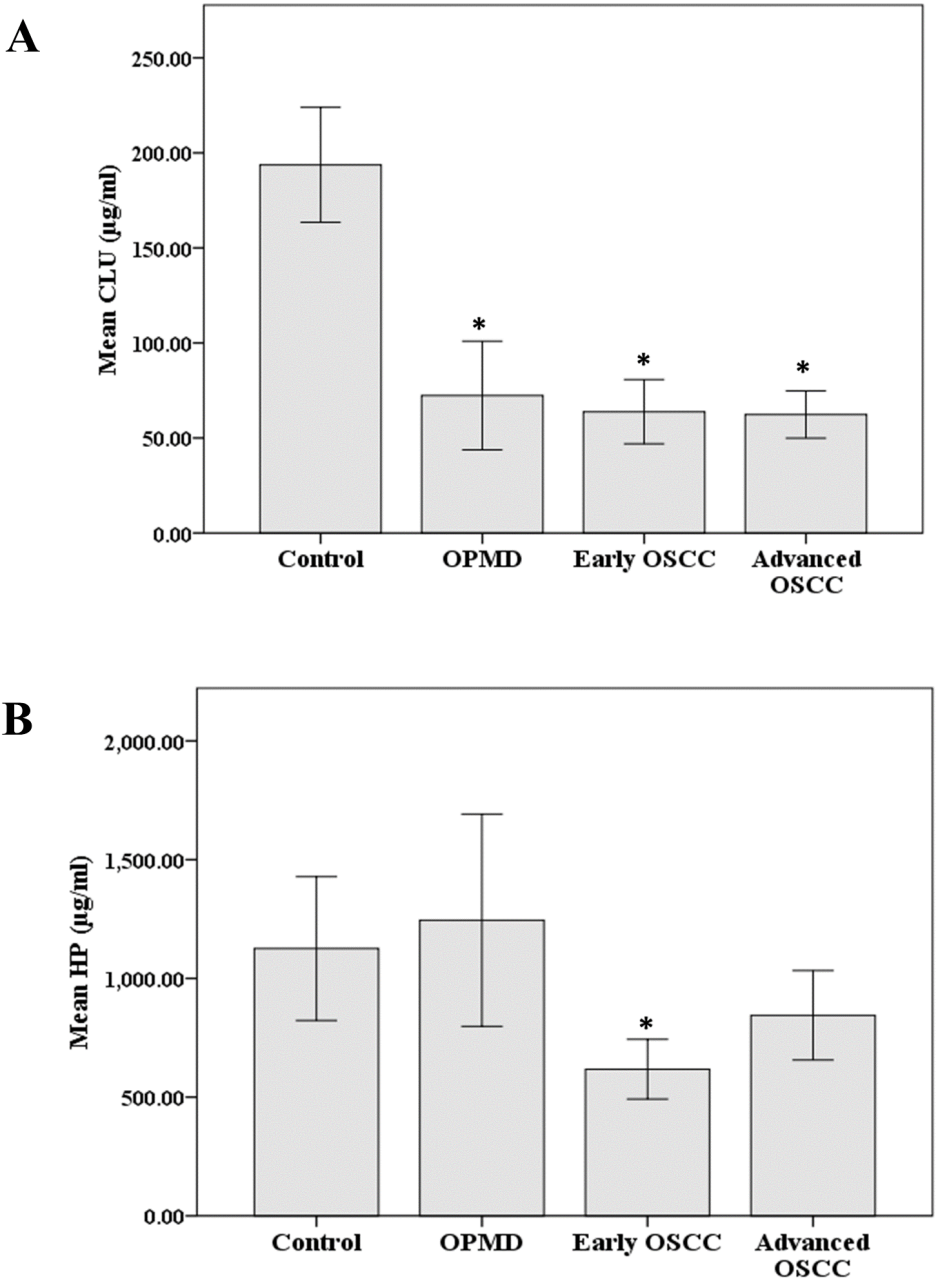
Expression of CLU and HP in ELISA analysis. The serum expression levels of (A) CLU and (B) HP in control, OPMD, early OSCC, and advanced OSCC. *p < 0.05, significant differences when compared with control.

Among the identified proteins, CLU and HP were selected for further validation, and the results are shown in Fig. 4. The mean serum levels of CLU in OPMD, early OSCC, and advanced OSCC were significantly lower than those in the control (*p* < 0.05) (Fig. 4A). Although only the mean serum level of HP in early OSCC was significantly lower compared to the control (*p* < 0.05), the concentration of HP also showed a lower level in advanced OSCC (Fig. 4B). Furthermore, we had performed receiver operator characteristic (ROC) analysis to evaluate the potential utility of serum CLU and HP in the detection of OSCC. The area under the (AUC) for CLU and HP was 0.945 (*p* < 0.001, 95% CI [0.91–0.99]) and 0.617 (*p* = 0.049, 95% CI [0.51–0.73]), respectively. The corresponding optimal cutoff values of CLU and HP to discriminate OSCC from control were 111.29 µg/ml (94.5% sensitivity, 89.0% specificity) and 1360.29 µg/ml (34.3% sensitivity, 90.4% specificity), respectively.

### IHC analysis

**Figure 5:**
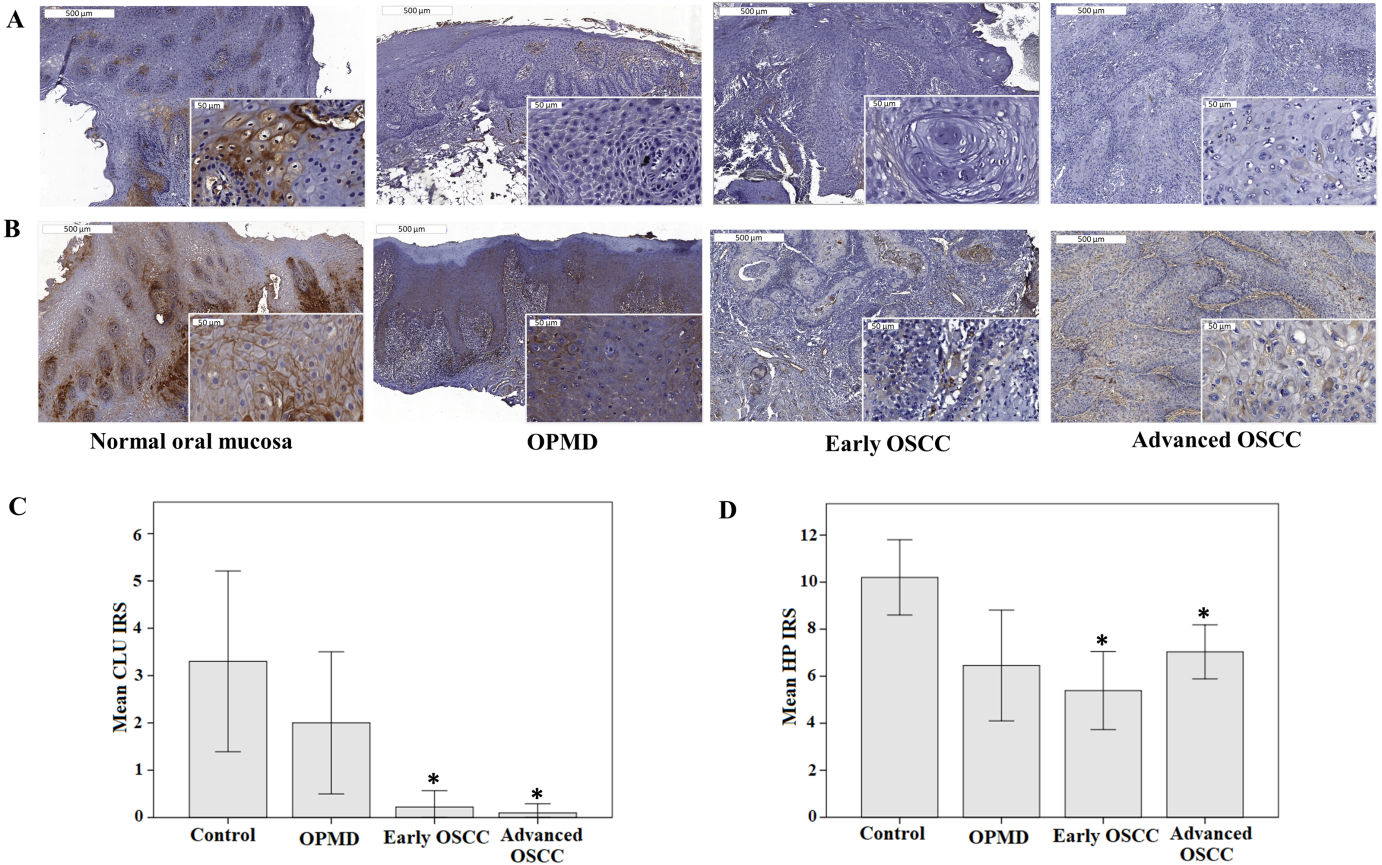
Expression of CLU and HP in IHC analysis. Representative immunostaining of (A) anti-CLU antibody and (B) anti-HP antibody in normal oral mucosa (control), OPMD, early OSCC, and advanced OSCC (Original magnification: 50×, 400× -inserts). The mean IRS of (C) CLU and (D) HP expression in control, OPMD, early OSCC, and advanced OSCC. *p < 0.05, significant differences when compared with control.

Immunohistochemical analysis revealed that the epithelial cells in control, OPMD, and OSCC tissues showed membranous and granular cytoplasmic staining of CLU and HP. Some OPMD and OSCC tissue samples displayed weak or no staining of CLU and HP (Figs. 5A–5B). We observed that the expressions of CLU and HP were significantly lower in early and advanced OSCC when compared with control (*p* < 0.05) (Figs. 5C–5D). In ROC analysis, the AUC for CLU and HP was 0.833 (*p* = 0.001, 95% CI [0.66–1.00]) and 0.804 (*p* = 0.003, 95% CI [0.66–0.95]), respectively. The optimal IRS cutoff for CLU and HP was 1.50 (70.0% sensitivity, 95.9% specificity) and 10.50 (60.0% sensitivity, 89.8% specificity), respectively. There were 54.5% OPMD and 95.9% OSCC patients had low CLU expression level. Similarly, 81.8% OPMD and 89.8% OSCC patients exhibited low HP expression levels as well. However, the expression levels of CLU and HP were not associated with the clinicopathological parameters of the disease.

## Discussion

In this study, the serum protein profiles of early OSCC, OPMD, and control were generated. Our proteomics data revealed 20 proteins that were differentially expressed in the serum of OPMD and OSCC when compared to controls. OPMDs were included in this study as OPMDs could manifest morphological alterations and might facilitate early arrest in the progression of OSCC. Several differentially expressed proteins that were detected in OSCC were also found to be differentially expressed in OPMD, which indicates the progression of oral carcinogenesis. We observed that dysregulation of several detected proteins, such as AMBP, C3, LRG1, and TF, are consistent with previous OSCC studies using different biological samples (Chen et al., 2014; Csosz et al., 2018; Jessie et al., 2013; Sekikawa et al., 2018). These proteins are involved in cell invasion, metastasis, signal transduction, and cell proliferation and may have an important role in oral carcinogenesis.

Based on the PANTHER analysis, most of the detected proteins were categorized according to their main biological functions. It is not surprising that biological regulation and metabolic process are the two most predominant biological processes. The biological regulations such as the regulation of angiogenesis, apoptosis, cell cycle, and homeostasis could influence the metabolic processes that involving cellular energy metabolism for the cancer cell to grow and proliferate during malignant transformation (Hanahan & Weinberg, 2011; Vander Heiden & DeBerardinis, 2017). Whereas most of the detected proteins were enriched within the extracellular space or region and reported as circulating blood microparticles in the functional annotation clustering analysis. The blood microparticles were associated with the promotion of angiogenesis, enhancement of metastatic potential, cellular survival, and evasion of immune surveillance during cancer progression (Gong et al., 2015). We also found that the activation of classical complement pathway was associated with the progression of OSCC (Chen et al., 2014; Gallenkamp et al., 2018). The activation of the classical complement pathway could induce angiogenesis, promoting cell proliferation and invasion, as well as modulate the immune response in cancer (Afshar-Kharghan, 2017). The serine-type endopeptidase activity was found to be associated with the progression of OSCC. The dysregulation of serine-type endopeptidase activity may lead to degradation of the extracellular matrix and imbalance of cellular homeostasis in cancers (Poddar, Maurya & Saxena, 2017). Additionally, other functional processes such as binding, catalytic, and transportation are known to be associated with carcinogenesis (Basten & Giles, 2013; Oruganty et al., 2020).

The IPA showed that multiple pathways and network functions are involved in the systemic response to OSCC, in which the LXR/RXR activation and acute phase response signaling pathways are the most prominent cellular pathways associated with OSCC. The activation of LXR/RXR pathway is involved in cholesterol and lipid metabolism, glucose production, and the regulation of immune responses. There is evidence showing that the LXR/RXR activation pathway is involved in many types of cancer (Lin & Gustafsson, 2015). The alterations in cholesterol and lipid metabolism have been associated with cancer development and progression (Borgquist et al., 2016). In line with the present findings, the activation of LXR was found to be involved in regulating the expression of ABCA1 and mediated the cholesterol efflux in OSCC (Kaneko et al., 2015). Furthermore, the activation of LXR was implicated in the modification of phosphoinositide 3-kinases (PI3K)/Akt transduction pathway, which plays a crucial role in cell proliferation, migration, and invasion during carcinogenesis (Dufour et al., 2013).

The activation of acute phase response signaling pathway in OSCC is in accordance with the observation that many cancers are associated with inflammation (Hanahan & Weinberg, 2011). It is believed that the activation of acute phase response could probably be due to the relationship among inflammation, innate immunity system, and cancer (Coussens & Werb, 2002). Moreover, inflammation could trigger cellular events and activate the immune response in the tumor microenvironment that can lead to malignant transformation of cells and carcinogenesis (Coussens & Werb, 2002; Landskron et al., 2014). The acute phase response is a systemic and non-specific reaction against any infection, inflammation, or trauma. It was found to be related to the modulation of the interleukin 6 (IL-6)/JAK/signal transducers and activators of transcription 3 (STAT3) signaling pathway, which is involved in the proliferation, progression, and metastasis of cancer cells (Johnson, O’Keefe & Grandis, 2018).

Of note, our results revealed 11 differentially expressed proteins (AAT, ALB, AMBP, APOA1, C3, CLU, HP, RBP4, SAMP, TF, and VDBP) that are associated with inflammation and immune responses in the IPA analysis. Furthermore, the detected proteins in this study with the majority are acute-phase proteins. The dysregulation of acute phase proteins has been found to be correlated with the progression of many cancers (Pang et al., 2010). These studies had revealed the potential role of acute phase proteins as cancer biomarkers due to the different expression pattern of acute phase proteins are observed in various cancers. On the other hand, our previous study had identified several host immune response-related proteins in the serum and samples of OSCC patients (Chen et al., 2014). A recent study reported that the activation of inflammatory cells especially neutrophils in the OSCC microenvironment could mediate cancer invasion and is associated with poor survival in various biological samples (Goertzen et al., 2018). Thus, oral chronic and/or acute inflammation is associated with the presence of immune cells and the complexity of the tumor microenvironment. Therefore, indirectly, the dysregulation of the detected proteins in this study might be related to the immune response associated with the development and progression of OSCC.

To ensure the association of the detected proteins in this study with OSCC, CLU and HP were selected for further validation using ELISA and IHC based on the bioinformatics analysis and literature search. These proteins are believed to have an essential role in the development and progression of OSCC. Protein CLU (apolipoprotein J) is a multifunctional secreted glycoprotein that present in various tissues and body fluids. It has been implicated in diverse biological processes, including cell adhesion, cell cycle regulation, lipid transportation, tissue remodeling and apoptosis, and immune system regulation. The expression of CLU was found to be decreased in OPMD and OSCC of both 2-DE and ELISA analyses. While in the IHC analysis, the expression of CLU was significantly lower in the OSCC tissues when compared with the control. Our study was in line with previous studies that show the decreased expression of CLU in the serum and saliva samples of OSCC patients (Chen et al., 2014; Hu et al., 2008). The decreased expression of CLU has also been reported in non-small-cell lung cancer, testicular seminoma, prostate, and esophageal cancers (Jin et al., 2017; Liu et al., 2013; Scaltriti et al., 2004; Zhang et al., 2003). These findings had indicated that CLU might present as a tumor suppressor in the early stage of carcinogenesis (Rizzi & Bettuzzi, 2010). Moreover, the decreased expression of CLU in the early onset of cancer progression may promote the activation of nuclear factor-*κ*B (NF-*κ*B) that is related to inflammatory processes and induce proliferation and invasion in advanced cancers (Bonacini et al., 2019).

Protein HP is a hemoglobin-binding protein that is primarily synthesized by the liver and released into circulation. The main functions of HP are to bind with free hemoglobin and prevent oxidative stress. The increased expression of HP is frequently detected in various cancers. Several studies had detected the increased expression of HP in the serum, plasma, and saliva samples of OSCC patients (Chen et al., 2014; Csosz et al., 2018; Jessie et al., 2013; Tung et al., 2013). However, our study showed opposite findings, in which the expression of HP was decreased in both serum and tissue samples of OPMD and OSCC. This could be due to the different isoforms of HP, the up-regulation of haptoglobin *α*2 chain and the down-regulation of haptoglobin *α*1 chain were reported in the serum samples of head and neck squamous cell cancer patients (Chen et al., 2008). This discrepancy may also present in other cancers such as liver and ovarian cancers (Sarvari et al., 2011; Tai et al., 2017; Zadeh Fakhar et al., 2019). Additionally, further validation through ELISA analysis in this present study showed a significantly lower level of HP in early OSCC. Similarly, a declining trend of HP was found in the plasma samples of OSCC patients with a significantly lower level of HP in the advanced stage (Chang et al., 2019). The current study also found a significantly lower expression of HP in early and advanced OSCC at the tissue level, which is in line with the previous study (Thiel et al., 2011). Thus, additional investigation into the involvement of HP in the development and progression of OSCC is needed.

In this study, several differentially expressed proteins in the serum samples of OPMD and OSCC have been detected using proteomics approach. These detected proteins might be related to inflammatory responses, which could likely indicate the development and progression of OSCC. However, this study has some limitations. The sample size of this study was relatively small, especially OPMDs due to the limited availability of the sample. The sensitivity of the validated proteins was not high. Nevertheless, high specificity might be a good check index for screening purposes because of the low possibility for false positive cases.

## Conclusions

In conclusion, our study has expanded the current understanding of the development and progression of OSCC. Our results had revealed that the majority of the significantly detected proteins are acute phase proteins, which were involved in the complex signaling pathway interactions in oral carcinogenesis. CLU and HP were found to be significantly decreased in early OSCC, which indicates that these proteins might have an impact in the early event of oral carcinogenesis. These proteins could be used as complementary biomarkers to improve the early detection of OSCC and may assist to predict the outcomes of OSCC patients. Further exploration in a larger cohort is warranted to assess the utility of these biomarkers in OSCC.

##  Supplemental Information

10.7717/peerj.11548/supp-1 Supplemental Information 1 Raw data from ELISA and IHC analyses

10.7717/peerj.11548/supp-2 Supplemental Information 2 Demographic and clinical characteristics of study samples (proteomics study)

10.7717/peerj.11548/supp-3 Supplemental Information 3 Demographic and clinical characteristics of study samples (validation study)

## Additional Information and Declarations

### Data Availability

The following information was supplied regarding data availability: Raw data from ELISA and IHC analyses are available in the Supplemental File. The data is available at MASCOT search engine and UniProtKB database: P01009, Q16352, P02768, P02760, P02647, Q8N726, P01024, P10909, P00738, P01877, P01859, P01834, P02750, P32119, Q92878, P02753, P02743, P02787, P18206, P02774
