# Supplementary material for: Comparative sera proteomics analysis of differentially expressed proteins in oral squamous cell carcinoma
Source: PeerJ. 2021 Jun 10;9:e11548. doi: 10.7717/peerj.11548 (PMC8199918; doi:10.7717/peerj.11548)
Supplement: Supplemental Information 2 [file peerj-09-11548-s002.docx]

**Supplementary Table 1**

Demographic and clinical characteristics of study samples (proteomics study)

|  | **Proteomics study (n = 60)** | | | |
| --- | --- | --- | --- | --- |
|  | **Control** | **OPMD** | **Early**  **OSCC** | **Advanced OSCC** |
| **N** | 10 | 10 | 20 | 20 |
| **Age (years)** | 55.70±16.21 | 53.80±14.87 | 57.90±15.76 | 63.10±12.10 |
| **Gender** |  |  |  |  |
| Male | 5 (50.0 %) | 1 (10.0 %) | 5 (25.0 %) | 6 (30.0 %) |
| Female | 5 (50.0 %) | 9 (90.0 %) | 15 (75.0 %) | 14 (70.0 %) |
| **Smoking** |  |  |  |  |
| No | 6 (60.0 %) | 9 (90.0 %) | 15 (75.0 %) | 16 (80.0 %) |
| Yes | 4 (40.0 %) | 1 (10.0 %) | 5 (15.0 %) | 4 (20.0 %) |
| **Drinking** |  |  |  |  |
| No | 9 (90.0 %) | 9 (90.0 %) | 14 (70.0 %) | 18 (90.0 %) |
| Yes | 1 (10.0 %) | 1 (10.0 %) | 6 (30.0 %) | 2 (10.0 %) |
| **Betel quid chewing** |  |  |  |  |
| No | 9 (90.0 %) | 6 (60.0 %) | 13 (65.0 %) | 10 (50.0 %) |
| Yes | 1 (10.0 %) | 4 (40.0 %) | 7 (35.0 %) | 10 (50.0 %) |
| **Site** |  |  |  |  |
| Tongue, floor of mouth | - | 1 (10.0 %) | 10 (50.0 %) | 8 (40.0 %) |
| Buccal mucosa, gingiva, lip & palate | - | 9 (90.0 %) | 10 (50.0%) | 12 (60.0 %) |
| **Histopathological diagnosis** | |  |  |  |
| Dysplasia | - | 2 (20.0%) | - | - |
| Non-dysplasia | - | 8 (80.0 %) | - | - |
| **Tumor size** |  |  |  |  |
| T1 & T2 | - | - | 20 (100.0 %) | 5 (25.0%) |
| T3 & T4 | - | - | 0 (0.0 %) | 15 (75.0%) |
| **Lymph node metastasis** |  |  |  |  |
| Negative | - | - | 20 (100.0 %) | 6 (30.0%) |
| Positive | - | - | 0 (0.0 %) | 14 (70.0%) |
| **Broders’ grading^‡^** |  |  |  |  |
| Well differentiated | - | - | 7 (43.7 %) | 8 (53.3 %) |
| Moderately & poorly differentiated | - | - | 9 (56.3 %) | 7 (46.7 %) |
