# Supplementary material for: Comparative sera proteomics analysis of differentially expressed proteins in oral squamous cell carcinoma
Source: PeerJ. 2021 Jun 10;9:e11548. doi: 10.7717/peerj.11548 (PMC8199918; doi:10.7717/peerj.11548)
Supplement: Supplemental Information 3 [file peerj-09-11548-s003.docx]

**Supplementary Table 2**

Demographic and clinical characteristics of study samples (validation study).

|  | **ELISA (n = 120)** | | | | **IHC (n = 70)** | | | |
| --- | --- | --- | --- | --- | --- | --- | --- | --- |
|  | **Control** | **OPMD** | **Early**  **OSCC** | **Advanced OSCC** | **Control** | **OPMD** | **Early**  **OSCC** | **Advanced OSCC** |
| **N** | 35 | 12 | 34 | 39 | 10 | 11 | 18 | 31 |
| **Age (years)** | 52.00±17.32 | 53.92±13.59 | 56.26±17.88 | 60.62±13.29 | 24.30±3.59 | 60.00±9.77 | 58.22±11.28 | 58.23±12.60 |
| **Gender** |  |  |  |  |  |  |  |  |
| Male | 18 (51.4 %) | 1 (8.3 %) | 12 (35.3 %) | 14 (35.9 %) | 3 (30.0 %) | 2 (18.2 %) | 5 (27.8 %) | 7 (22.6 %) |
| Female | 17 (48.6 %) | 11 (91.7 %) | 22 (64.7 %) | 25 (64.1 %) | 7 (70.0 %) | 9 (81.8 %) | 13 (83.3 %) | 24 (77.4 %) |
| **Smoking^‡^** |  |  |  |  |  |  |  |  |
| No | 26 (74.3 %) | 11 (91.7 %) | 24 (70.6 %) | 27 (69.2 %) | 7 (87.5 %) | 9 (90.0 %) | 15 (83.3 %) | 27 (87.1 %) |
| Yes | 9 (25.7 %) | 1 (8.3 %) | 10 (29.4 %) | 12 (30.8 %) | 1 (12.5 %) | 1 (10.0 %) | 3 (16.7 %) | 4 (12.9 %) |
| **Drinking^‡^** |  |  |  |  |  |  |  |  |
| No | 33 (94.3 %) | 11 (91.7 %) | 26 (76.5 %) | 32 (82.1 %) | 7 (87.5 %) | 9 (90.0 %) | 12 (66.7 %) | 19 (61.3 %) |
| Yes | 2 (5.7 %) | 1 (8.3 %) | 8 (23.5 %) | 7 (17.9 %) | 1 (12.5 %) | 1 (10.0 %) | 6 (33.3 %) | 12 (38.7 %) |
| **Betel quid chewing^‡^** |  |  |  |  |  |  |  |  |
| No | 32 (91.4 %) | 8 (66.7 %) | 24 (70.6 %) | 23 (59.0 %) | 8 (100.0 %) | 6 (60.0 %) | 6 (33.3 %) | 8 (25.8 %) |
| Yes | 3 (8.6 %) | 4 (33.3 %) | 10 (29.4 %) | 16 (41.0 %) | 0 (0.0 %) | 4 (40.0 %) | 12 (66.7 %) | 23 (74.2 %) |
| **Site** |  |  |  |  |  |  |  |  |
| Tongue, floor of mouth | - | 2 (16.7 %) | 19 (55.9 %) | 21 (53.8 %) | - | 2 (18.2 %) | 4 (22.2 %) | 8 (25.8 %) |
| Buccal mucosa, gingiva, lip & palate | - | 10 (83.3 %) | 15 (44.1 %) | 18 (46.2 %) | 10 (100.0 %) | 9 (81.8 %) | 14 (77.8 %) | 23 (74.2 %) |
| **Histopathological diagnosis** | |  |  |  |  |  |  |  |
| Dysplasia | - | 2 (16.7 %) | - | - | - | 5 (45.5 %) | - | - |
| Non-dysplasia | - | 10 (83.3 %) | - | - | - | 6 (54.5 %) | - | - |
| **Tumor size** |  |  |  |  |  |  |  |  |
| T1 & T2 | - | - | 34 (100.0 %) | 13 (33.3 %) | - | - | 18 (100.0 %) | 10 (32.3 %) |
| T3 & T4 | - | - | 0 (0.0 %) | 26 (66.7 %) | - | - | 0 (0.0 %) | 21 (67.7 %) |
|  |  |  |  |  |  |  |  |  |
| Supplementary Table 1 continued | | |  |  |  |  |  |  |
|  | **Control** | **OPMD** | **Early**  **OSCC** | **Advanced OSCC** | **Control** | **OPMD** | **Early**  **OSCC** | **Advanced OSCC** |
| **Lymph node metastasis** |  |  |  |  |  |  |  |  |
| Negative | - | - | 34 (100.0 %) | 9 (23.1 %) | - | - | 18 (100.0 %) | 8 (25.8 %) |
| Positive | - | - | 0 (0.0 %) | 30 (76.9 %) | - | - | 0 (0.0 %) | 23 (74.2 %) |
| **Broders’ grading^‡^** |  |  |  |  |  |  |  |  |
| Well differentiated | - | - | 12 (44.4 %) | 18 (62.1 %) | - | - | 6 (33.3 %) | 5 (16.1 %) |
| Moderately & poorly differentiated | - | - | 15 (55.6 %) | 11 (37.9 %) | - | - | 12 (66.7 %) | 26 (83.9 %) |

^‡^ Data missing
